# Assessment of Pertussis Vaccine Protective Effectiveness in Children in the Amhara Regional State, Ethiopia

**DOI:** 10.1155/2020/8845835

**Published:** 2020-10-13

**Authors:** Solomon Taye, Belay Tessema, Baye Gelaw, Feleke Moges

**Affiliations:** ^1^Department of Medical Microbiology, School of Biomedical and Laboratory Sciences, College of Medicine and Health Sciences, University of Gondar, P.O. Box 196, Gondar, Amhara Regional State, Ethiopia; ^2^Department of Medical Laboratory Sciences, College of Medicine and Health Sciences, Wachemo University, P.O. Box 667, Hawassa, South Nations Nationalities and Peoples Regional State, Ethiopia

## Abstract

**Background:**

*Bordetella pertussis* is a human pathogen which causes pertussis, or whooping cough. The diphtheria-tetanus-pertussis immunization has significantly reduced the morbidity and mortality of pertussis globally. However, higher prevalence and resurgence of pertussis cases among both vaccinated and unvaccinated people has raised questions on the effectiveness of pertussis vaccine over time. Therefore, the objective of this study was to assess the protective effectiveness of pertussis vaccine in the Amhara Regional State, Ethiopia.

**Methods:**

A nested matched case-control study design approach was used with vaccinated individuals as cases and unvaccinated individuals as controls. The study was conducted from July 2018 to February 2019. Real-time (RT-) PCR assay was done to ascertain the presence of pertussis among clinically suspected patients. Bivariable and multivariable logistic regression analyses were computed to estimate the crude and adjusted odds ratios (ORs), respectively. Vaccine effectiveness was calculated as (1 − OR) × 100. Adjusted OR with 95% CI and a *P* value <0.05 were used to assess statistical significance.

**Results:**

A total of 112 vaccinated and 223 unvaccinated controls were enrolled for the study. Of the total participants, 173/335 (51.6%) were males. The prevalence of pertussis among vaccinated was 35/112 (31.3%), whereas it was 84/223 (37.7%) among the control group. The adjusted matched vaccine protective effectiveness against *B. pertussis* infection following three doses of whole-cell vaccine was 25% among children aged between 6 and 9 years. Adjusted estimates of vaccine protective effectiveness for participants who had complete vaccination, stratified by time since last vaccination, were 50% at 6 years, 34% at 7 years, and 2% at 8–9 years since last vaccination.

**Conclusion:**

Despite the availability and good coverage of childhood vaccination, the effectiveness of pertussis vaccine was found to be low in the Amhara region, Ethiopia. Moreover, we observed declining trends in the protective effectiveness of the vaccine after 6 years of vaccination. Thus, by considering the waning nature of immune response which is induced by whole-cell vaccine during early life, booster dose is highly recommended to optimize pertussis prevention and control strategies.

## 1. Introduction

Pertussis, or whooping cough, is a highly contagious respiratory tract infection which has been poorly controlled compared with other vaccine-preventable diseases [1, 2]. Globally, there were an estimated 24.1 million pertussis cases and 160,700 deaths from pertussis in children younger than 5 years in 2014, with the African region contributing the largest proportions of 7.8 million (33%) cases and 92,500 (58%) deaths. Of this, an estimated 5.1 million (21%) pertussis cases and 85,900 (53%) deaths were among infants younger than 1 year. Even in countries where pertussis vaccination is widely available, there is still a significant burden of pertussis disease [2–4].

Mass vaccination programs for childhood diseases have been highly successful in reducing the incidence and public health impact of the targeted diseases. The whole-cell pertussis (wP) vaccine introduced in the 1940s was replaced by the less reactogenic acellular (aP) vaccines in the1980s. The diphtheria-tetanus-pertussis (DTP) immunization has significantly reduced the morbidity and mortality of pertussis globally. Thus, DTP vaccination has made a substantial impact on the pertussis epidemiology. However, there is still significant infant morbidity and mortality due to pertussis across the globe especially in developing countries [4, 5].

Childhood pertussis vaccination has a limited duration of protection and therefore raises concerns on its effectiveness. In France, the effectiveness of wP vaccines appeared to remain unchanged for more than 30 years, and similarly, in Australia, the effectiveness of wP vaccine was estimated to be 91%. In USA, the effectiveness of wP vaccine was estimated to be 76% and the effectiveness of one dose of aP vaccine was approximately 50%. In Poland, for reasons unknown, the effectiveness decreased between 1996 and 2001 from 97.3% to 73.5%, and a similar reduction in the Netherlands was also noted [6–8].

In Austria, the effectiveness of a three-dose course of wP vaccine for the prevention of pertussis hospitalization was estimated to be 79% when compared to 92% after a three-dose course of aP vaccines. A similar decrease in hospitalization after changing from wP to aP vaccines was observed in Canada. By contrast, a study in rural Senegal reported that wP vaccines were more effective (67%) than a two-component aP vaccine (32%). In Germany, even one dose of vaccine was 68% effective in reducing hospitalization in infants [6–9].

Although the effectiveness of pertussis vaccine remains high, a number of factors can affect vaccine effectiveness. These include vaccine transportation, vaccine dose, vaccination schedule (interval), number of vaccinations, type of strain used to produce vaccine, vaccine type (attenuated or killed), vaccine component (one component, two components, etc.), perceived seriousness of the disease, perceived safety and efficacy of the vaccine, social pressure, educational status of the community, nonresponders, and ethnicity or genetics of the individual. As a consequence, due to the limited duration of protection, many countries recommend a preschool and/or an adolescent booster dose [6, 10, 11].

In Ethiopia, wP vaccine is given at 6, 10, and 14 weeks of life in combination with diphtheria and tetanus according to the recommended national vaccination guideline. However, pertussis booster vaccination is not included in the national vaccination strategy of Ethiopia. Despite the good coverage of pertussis vaccination in Ethiopia (85% for 1 dose of DTP and 72% for 3 doses of DTP) [12], several outbreaks of pertussis have recently been reported from several localities. These outbreaks were based on clinical diagnosis and not confirmed by laboratory tests. Nevertheless, there are questions whether these outbreaks are due to the current wP vaccine becoming less effective, or due to reduced vaccine coverage which allows the bacteria to spread among fully or partially vaccinated populations [5, 13].

According to the federal Ministry of Health (FMOH) Ethiopia, in 2016, there were 4,719 clinically confirmed pertussis cases with 9 deaths. In the Amhara Regional State, there were 351 clinically confirmed pertussis cases and 3 deaths reported in the same year [unpublished report from the FMOH Ethiopia, 2017].

Postlicensure real-world evaluation of vaccine implementation is important for establishing evidence of vaccine effectiveness and program impact, including indirect effects. Furthermore, evaluating the direct protective effects of vaccines in the individuals who were vaccinated was also important. These, postlicensure protective effectiveness evaluations are crucial for policy makers to assess vaccine roll-out, highlight issues in program implementation, and determine the total impact of direct and indirect effects at population level [13, 14]. In this regard, there are no studies assessing the protective effectiveness of pertussis vaccine in the study area and Ethiopia at large. Therefore, the aim of the present study was to determine the protective effectiveness of pertussis vaccine in the Amhara Regional State, Ethiopia.

## 2. Materials and Methods

### 2.1. Study Area and Period

The study was conducted in the Amhara Regional State, the second most populous region in Ethiopia. The region is divided into 10 zones and 3 administrative cities, with a population of 21.5 million [15, 16]. In the region, there are 69 government hospitals. For this study, six hospitals were selected based on convenience and laboratory infrastructure. The University of Gondar comprehensive specialized hospital, Felege-Hiwot comprehensive specialized hospital in Bahir Dar town, Ebnat hospital in Ebnat Woreda (South Gondar Zone), Masha hospital in Mekdela Woreda (South Wollo Zone), and Debark and Janamora hospitals (both in North Gondar Zone) were selected for this study. The study was conducted from July 2018 to February 2019.

### 2.2. Ethical Clearance

Ethical approval was obtained from the University of Gondar Institutional Review Board (IRB) (Ref. no. O/V/P/RCS/05/376/2017). Each hospital, laboratory, and the outpatient department were communicated through written letter obtained from the University of Gondar IRB. Each study subject was informed about the objectives of the study that would contribute towards the understanding of pertussis vaccine effectiveness. The study subjects were informed that it is their right not to participate or withdraw from the study at any time during the study. An assent form by parents or legal guardians was completed for children. For each clinically suspected case, the responsible clinician of the patient was informed, and treatment was initiated as per national guidelines. Data taken from each study subject was numerically coded, and results obtained were used only for study purposes and kept confidential.

### 2.3. Study Design and Subjects

Vaccine protective effectiveness is the percent reduction in the frequency of pertussis disease among vaccinated (3 doses) people compared to people not vaccinated. In this study, vaccine protective effectiveness (VE) was estimated by a nested matched case-control study design among children aged 6–9 years old. This study was nested in a larger cohort study investigating the prevalence of pertussis in the Amhara Regional State in Ethiopia. Vaccine protective effectiveness was estimated among laboratory confirmed pertussis infections between vaccinated participants and unvaccinated controls with comparable age groups. The Expanded Program on Immunization (EPI) registration in the hospitals was used to determine whether children were vaccinated for pertussis vaccination or not. From July 2018 to February 2019, child participants meeting the CDC clinical case definition for pertussis [4] and who visited any of the hospitals were recruited.

The clinical case definition for pertussis is a patient with cough illness lasting ≥2 weeks, with at least one of the following signs or symptoms, paroxysms of coughing, inspiratory whoop, posttussive vomiting without other apparent cause, or apnea (with or without cyanosis) (for infants <1 year old only). All children aged between 6 and 9 years old with clinically suspected pertussis were included in the study. Participants started on antibiotic treatment, partially vaccinated, and those where vaccination status was unknown or incomplete were excluded from the study. Laboratory confirmation of pertussis infection was conducted using an RT-PCR assay using methods described by Roorda et al. [17]. Vaccinated PCR-positive and PCR-negative children were assigned as study subjects (vaccinated group) whereas unvaccinated PCR-positive and PCR-negative children were assigned as unvaccinated control group.

Clinical and sociodemographic features of each patient were recorded by a physician and a nurse. Sociodemographic characteristics such as age, gender, residence, educational status; vaccine-related characteristics such as vaccination status, number of vaccinations, time since last vaccination, and clinical features such as cough ≥14 days, coughing paroxysms, respiratory distress, whoops, apnea, cyanosis, syncope, and posttussive vomiting were assessed and determined [4, 8, 14].

### 2.4. Sample Size Determination

Two population proportion formulas were used to calculate the required sample size. The Epi Info software (matched case-control) method with a *p*1(66.7%), *p*2(50%), *r*(2), OR(2), *Zα*/_2_(95%), and *Z*_1−*β*_(80%) was used to calculate the sample size. Accordingly, *n*_1_ was 112 vaccinated PCR-positive and -negative cases, whereas *n*_2_ was 223 unvaccinated PCR-positive and -negative controls giving a total of 335 study participants.

### 2.5. Nasopharyngeal Swab Collection and Processing

Nasopharyngeal swabs were collected by trained and experienced physicians and nurses from all the 335 study subjects using Dacron swabs (COPAN, FLOQSwaps Technologies, Brescia, Italy). Samples were transported in a cold container with dry ice to the microbiology laboratory of each of the hospitals for analysis and stored at −20°C until DNA extraction was conducted.

### 2.6. DNA Extraction

DNA extraction was performed at the University of Gondar Medical Microbiology laboratory. DNA was directly extracted from nasopharyngeal swabs using a commercial DNA extraction kit (QIAamp Mini Kit, Qiagen, Hilden, Germany) according to the manufacturer's instructions. The DNA was eluted into 100 *μ*L volume elution buffer and the extracted DNA was stored at −80°C until PCR amplification. All 335 extracted DNA samples were transported to the Division of Medical Microbiology, University of Cape Town (UCT), South Africa, for RT-PCR assay.

### 2.7. Multiplex RT-PCR Assay

Detection of *Bordetella* species was performed using multiplex RT-PCR assays for the amplification of the insertion sequences (IS)*481* and *1002* modified from Roorda et al. [17]. DNA was amplified with the CFX96® thermal RT-PCR System (Bio-Rad). The PCR protocol used was as follows: at 50°C for 2 minutes, 95°C for 10 minutes; followed by 45 cycles of 95°C for 15 seconds, 60°C for 1 minute. A positive result was defined as a cycle threshold (Ct) value below 38 cycles. A Ct value of >38 or no detection was considered negative. The final interpretation for PCR result was as follows: *B. pertussis* (Ct < 38 for both IS*481* and IS*1002*), *B. parapertussis* (Ct < 38 for IS*1002* and > 38 or no amplification (N/A) for IS*481*), probable *B*. *bronchiseptica/holmesii* (Ct < 38 for IS*481* and N/A for IS*1002*) and probable *B*. *bronchiseptica/holmesii* but could not exclude low level of *B. pertussis* (Ct < 38 for IS*481* and > 38 for IS*1002*).

No-template controls were included in each PCR run as a negative control. A laboratory-prepared and sequence-confirmed control vector using known reference strains of *B. pertussis* and *B. parapertussis* was used as positive control. Furthermore, an internal amplification control using an internal fragment of the green fluorescent protein (GFP) was used to monitor possible reaction inhibition during PCR amplification [18].

### 2.8. Covariates

We included important demographic information for all cases and controls such as age, sex, religion, educational status, family educational status, family occupation, family size, and residence address. We have also assessed chronic disease conditions including chronic respiratory diseases. These datasets were used and analyzed.

### 2.9. Data Analysis

The baseline characteristics of the study population were summarized using median and frequencies. Crude and adjusted ORs were computed using bivariable and multivariable logistic regression analyses, respectively. In this study, children with pertussis disease (cases) were ascertained through PCR assay, and two appropriate controls were selected for each case. Vaccination status was determined for the cases and controls. Vaccine effectiveness was determined using the appropriate formula and applying the rare-disease assumption to substitute odds ratio (OR) for relative risk (RR), since the OR is an estimate of the RR. Analysis of VE against *Bordetella* infection was calculated as 1 − OR (1 − OR) × 100. A *P* value <0.05 was used to assess statistical significance.

## 3. Results

### 3.1. Sociodemographic Characteristics

In this study, 112 vaccinated and 223 unvaccinated controls with a total number of 335 study participants were included. All vaccinatedparticipants received the same type and recommended doses of pertussisvaccination. The age of the study participants ranged from 6 to 9 years with median age of 8 years, and 173/335 (51.6%) of the participants were male. One hundred eighty-nine of the study participants (56.4%) were rural residents, 156/335 (46.6%) of them had six and more family members, and 250/335 (74.6%) of the study participants were school grades 1–4. Approximately half of the study participant (49.9%) families were farmers by occupation (Table 1).

### 3.2. Vaccine Protective Effectiveness

The prevalence of pertussis in the vaccinated participants was 35/112 (31.3%) while the prevalence of pertussis in the unvaccinated control group was 84/223 (37.7%). In the present study, the matched VE against pertussis infection following three doses of whole-cell vaccine was 25% (95% CI: 20.6%–30.8%) among children aged between 6 and 9 years (Table 2).

The characteristics of PCR confirmed pertussis among vaccinated and unvaccinated controls are shown in Table 3. High numbers of pertussis positive participants were observed in the vaccinated participants at Masha and Ebnat hospitals, while the least pertussis positive participants were found in Janamora hospital. On the other hand, higher numbers of pertussis positive participants were observed among the unvaccinated group at University of Gondar and Ebnat hospitals, whereas the least pertussis positives among unvaccinated group were found in Felege-Hiwot hospital. Relatively higher numbers of pertussis positives were detected among children participants aged 9 years old in both vaccinated and unvaccinated groups followed by 8-year-olds.

Compared with unvaccinated participants, the adjusted estimates of pertussis VE among study participants who had complete vaccination, stratified by time since last vaccination were 50% (95%, CI: 42.8%–56.6%) at 6 years, 34% (95% CI: 28.6%–40.4%) at 7 years, and 2% (95% CI: 1.2%–3.4%) at 8–9 years. Adjusted pertussis VE decreased over time by 16% per year since last vaccination among participants with complete vaccination with a sharp decrease of approximately 32% at 8–9 years. On the other hand, the proportion of pertussis positive cases was increased by at least 3% per year since last vaccination (Table 4).

## 4. Discussion

Vaccine effectiveness is a “real-world” view of how a vaccine (which may have already proven to have high vaccine efficacy) reduces disease in a population [14]. This measure can assess the net balance of benefits and adverse effects of a vaccination program, not just the vaccine itself, under more natural field conditions rather than in a controlled clinical trial. Vaccine effectiveness is proportional to vaccine potency (i.e., vaccine efficacy) but is also affected by how well target groups in the population are immunized, which itself may reflect difficulties in maintaining proper storage conditions of a vaccine, such as the cold chain, access to health care, and vaccine cost, as well as by other non–vaccine-related factors that influence the real-world outcomes of hospitalizations, ambulatory visits, or costs [14, 19].

Pertussis resurgences around the world are a major challenge for immunization programs [10, 20, 21, 22]. To the best of our knowledge, protective effectiveness of pertussis vaccine has not been reported in Ethiopia. In this study, vaccine protective effectiveness for completed vaccination (3 doses) was generally low (25%). Our finding was lower than wP VE studies conducted in 90% in USA [10], 57% in Canada [20], and 55% in Senegal [23]. However, contrary to our finding, there is evidence that receiving wP at least the first dose of pertussis-containing vaccine provides greater and more long-lived protection, irrespective of the nature of subsequent doses [22]. The differences in VE these studies might be due to the sample size difference.

Individuals who receive wP vaccine had been shown to exhibit significant waning of immunity [1, 10, 21, 22, 24]. Our results showed similar rapid waning of immunity. We observed better protective effectiveness of the pertussis vaccine up to 6 years of age compared with after 6 years of age. Moreover, this study showed increased evidence of waning of immunity within 8-9 years. For each year that elapsed from wP vaccination, there was a 3% increment of testing positive for pertussis among fully vaccinated group. Our findings were in line with the studies conducted in Canada which showed that low vaccine effectiveness beyond 4 years from last vaccination is attributable to waning immunity [1].

In our study, the wP VE was also lower compared with aP studies conducted in 62% in Canada [1], 93% in New Zealand [25], 99.8% in Germany [19], and 53% in USA [24]. However, our finding of wP VE was similar to the aP VE study conducted in 31% in Senegal [23]. Waning of immunity in individuals who receive aP vaccine is also shown [1]. A recent meta-analysis identified increased odds of pertussis of 33% for each year following the DTaP dose. That meta-analysis supports our finding that beyond 7 years since the last vaccination there is minimal protective effect of the vaccine, despite some evidence showing persistent humoural immunity [1, 7, 19].

Immunization advisory committees in Western countries recommend a booster for adolescents and adults for those vaccinated with aP vaccine [1, 14, 19, 21, 22, 25]. However, for most African countries which use wP, vaccine booster dose was not included in their national immunization programs. Absence of booster dose may have contributed to several of the outbreaks of pertussis in Africa including Ethiopia.

Many researches had indicated the immune response induced by full dose of wP vaccine starts to wane significantly after 4 years [14, 25]. In Ethiopia, fully vaccinated is defined as 3 doses of wP if the third dose was administered on 14 weeks of life. In Ethiopia, due to the absence of studies showing the interval and the best age for booster dose, it is hard to recommend the best age and interval for booster vaccination. Thus, various studies must be conducted at different age groups to determine the optimal age and interval for booster dose. CDC defines fully vaccinated as 5 doses of DTaP or 4 doses of DTaP if the fourth dose was administered on or after the fourth birthday. Thus, the CDC routinely recommends DTaP at 2, 4, and 6 months, at 15 through 18 months, and at 4 through 6 years. In USA, the Tdap booster dose starts as a single dose for those 11 through 18 years of age with preferred administration at 11 through 12 years of age. For countries which use aP vaccine, a booster dose also recommended for adults 65 years and older [4, 12].

Public health surveillance provides a scientific and factual database essential to informed decision making and appropriate public health action. The key objective of surveillance is to provide information to guide interventions. The public health objectives and actions needed to make successful interventions determine the design and implementation of surveillance systems [26]. The ultimate objective of surveillance is to prevent the spread of pertussis epidemics, and, thus, the ministry of health needs to intervene quickly to stop the spread of disease. Therefore, the surveillance system should provide laboratory-confirmed early warning information. A good surveillance study should provide a series of demographic and health surveys including the most affected age groups for priority purposes. Thus, in addition to conducting studies on the importance of booster dose, it is also important to conduct laboratory-assisted surveillance studies at different localities of Ethiopia. Conducting different surveillance studies will help policy makers and stake holders for the better prevention and control of the disease [4, 12, 26].

The strengths of this study included the use of two unvaccinated for each vaccinated participant to estimate the effectiveness of wP vaccine. The use of two controls adds to our confidence calculating the effectiveness of the vaccine by decreasing bias from one to one assignment of case and control. Secondly, the use of PCR for case ascertainment, compared to culture, has helped to assign the participants as pertussis positive and negative more accurately.

This study had certain limitations. Firstly, this study enrolled children aged 6–9 years old and, thus, the findings of the study might not accurately reflect the exact VE because vaccine-induced immunity wanes as the children grew. Secondly, we enrolled a relatively small sample size and this might affect generalization of the finding to the general population.

## 5. Conclusion

Despite the good pertussis vaccination coverage, in the Amhara Regional State, this study showed that the adjusted VE was low (25%). Moreover, we observed declining trends in the protective effectiveness of the vaccine after 6 years of vaccination. Our findings suggest the need to revise our strategy for pertussis vaccination and highlight the importance of booster dose in the national vaccination program in Ethiopia. The benefit of additional boosters likely varies based on the cause of pertussis resurgence, which suggests that a better understanding of the current epidemiology of pertussis is critical for optimizing public policy. In addition, further large-scale, multiregional pertussis VE studies should be conducted in the country in order to revise the current vaccination program and possibly to incorporate a booster dose for effective prevention and control of the disease.

## Figures and Tables

**Table 1:** Socio-demographic characteristics of vaccinated and un-vaccinated controls, matched by age, Amhara Regional State, Ethiopia, 2019.

Characteristics	Category	Vaccinated, *n* (%)	Un-vaccinated, controls *n* (%)	*P* value
		112 (100)	223 (100)	
Gender	Male	56 (50.0)	117 (52.5)	0.89
	Female	56 (50.0)	106 (47.5)

Age (years)	6	31 (30.4)	49 (22.0)	0.67
	7	27 (25.9)	55 (24.7)
	8	26 (18.8)	63 (28.3)
	9	28 (25.0)	56 (25.1)

Residence	Rural	60 (53.6)	129 (57.8)	0.64
	Urban	52 (46.4)	94 (42.2)

Religion	Orthodox	83 (74.1)	179 (80.3)	0.83
	Muslim	26 (23.2)	39 (17.5)
	Protestant	3 (2.7)	5 (2.2)

Educational status of participants	Illiterate	30 (26.8)	55 (24.7)	0.85
	Grade 1–4	82 (73.2)	168 (75.3)

Mother educational status	Illiterate	51 (45.5)	110 (49.3)	0.66
	Read and write	8 (7.1)	18 (8.1)
	Grade 1–8	28 (25.0)	52 (23.3)
	Grade 9–12	15 (13.4)	24 (10.8)
	Grade 12+	10 (8.9)	19 (8.5)

Father educational status	Illiterate	42 (37.5)	76 (34.1)	0.71
	Read and write	8 (7.1)	12 (5.4)
	Grade 1–8	16 (14.3)	58 (26.0)
	Grade 9–12	19 (17.0)	37 (16.6)
	Grade 12+	27 (24.1)	40 (17.9)

Family size	<4 people	23 (20.5)	52 (23.3)	0.69
	5 people	36 (32.1)	68 (30.5)
	>6 people	53 (47.3)	103 (46.2)

Family occupation	Governmental	24 (21.4)	29 (13.0)	0.99
	Non-governmental	4 (3.6)	8 (3.6)
	Merchant	28 (25.0)	63 (28.3)
	Farmer	51 (45.5)	116 (52.0)
	Student	0 (0.0)	3 (1.3)
	Unemployed	2 (1.8)	2 (0.9)
	Others	3 (2.7)	2 (0.9)

**Table 2:** Frequency and percentage of pertussis in vaccinated and unvaccinated control groups, Amhara Regional State, Ethiopia, 2019.

Category	Total participants, *N* (%)	Positives, *N* (%)	Negatives, *N* (%)
Vaccinated (cases)	112 (33.4)	35 (29.4)	77 (35.6)
Unvaccinated (controls)	223 (66.6)	84 (70.6)	139 (64.4)
Total	335 (100.0)	119 (100.0)	216 (100.0)

*N* = number.

**Table 3:** Distribution of pertussis positives among vaccinated and un-vaccinated control groups in different hospitals and socio-demographic variables, Amhara Regional State, Ethiopia, 2019.

Characteristics		Vaccinated		Un-vaccinated		Total Infected *N* (%)	*P* value
		Total	Infected, *N* (%)	Total	Infected, *N* (%)		
Gender	Male	56	15 (42.9)	117	40 (47.6)	55 (46.2)	0.86
	Female	56	20 (57.1)	106	44 (52.4)	64 (53.8)

Age (years)	6	31	6 (17.1)	49	16 (19.0)	22 (18.5)	0.74
	7	27	7 (20.0)	55	19 (22.6)	26 (21.8)
	8	26	10 (28.6)	63	24 (28.6)	34 (28.6)
	9	28	12 (34.3)	56	25 (29.8)	37 (31.1)

Residence	Rural	60	24 (68.6)	129	46 (54.8)	70 (58.8)	0.79
	Urban	52	11 (31.4)	94	38 (45.2)	49 (41.2)

Family size	<4	23	5 (14.3)	52	22 (26.2)	27 (22.7)	0.68
	5	36	9 (25.7)	68	26 (31.0)	35 (29.4)
	>6	53	21 (60.0)	103	36 (42.9)	57 (47.9)

Hospital	UoG	19	4 (11.4)	38	24 (28.6)	28 (23.5)	0.92
	FH	19	2 (5.7)	38	3 (3.6)	5 (4.2)
	Masha	18	13 (37.1)	36	14 (16.7)	27 (22.7)
	Debark	19	4 (11.4)	38	6 (7.1)	10 (8.4)
	Janamora	18	1 (2.9)	35	15 (17.9)	16 (13.4)
	Ebnat	19	11 (31.4)	38	22 (26.2)	33 (27.7)

**Table 4:** Adjusted estimates of pertussis vaccine protective effectiveness among different age categories.

Variable		Vaccinated	Un-vaccinated	Adjusted vaccine effectiveness, % (95% CI)	*P* value
		pertussis positive/negative	pertussis positive/negative		
Age (years)	6	6/25	16/33	50 (42.8–56.6)	0.65
	7	7/20	19/36	34 (28.6–40.4)
	8-9	22/32	49/70	2 (1.2–3.4)

## Data Availability

The original data for this study are available from the corresponding author upon request.

## Abbreviations

aP: Acellular pertussis
Ct: Cycle threshold
DTP: Diphtheria-tetanus-pertussis
EPI: Expanded Program on Immunization
FMOH: Federal Ministry of Health
GFP: Green fluorescent protein
IRB: Institutional Review Board
IS: Insertion sequences
OR: Odds ratio
PCR: Polymerase chain reaction
RR: Relative risk
UCT: University of Cape Town
VE: Vaccine effectiveness
wP: Whole-cell pertussis.

## References

[1] Schwartz K. L., Kwong J. C., Deeks S. L. (2016). Effectiveness of pertussis vaccination and duration of immunity. *CMAJ*.

[2] Paddock C. D., Sanden G. N., Cherry J. D. (2008). Pathology and pathogenesis of FatalBordetella pertussisInfection in infants. *Clinical Infectious Diseases*.

[3] Yeung K. H. T., Duclos P., Nelson E. A. S., Hutubessy R. C. W. (2017). An update of the global burden of pertussis in children younger than 5 years: a modelling study. *The Lancet Infectious Diseases Lancet*.

[4] Centers for Disease Control and Prevention (2015). *Epidemiology and Prevention of Vaccine-Preventable Diseases: Pertussis*.

[5] Wagner B., Melzer H., Freymüller G. (2015). Genetic variation of *Bordetella pertussis* in Austria. *PLoS One*.

[6] Mooi F. R., Van-Loo I. H., King A. J. (2001). Adaptation of *Bordetella pertussis* to vaccination: a cause for its reemergence?. *Emerging Infectious Diseases*.

[7] Niewiesk S. (2014). Maternal antibodies: clinical significance, mechanism of interference with immune responses, and possible vaccination strategies. *Frontiers in Immunology*.

[8] Rendi-Wagner P., Paulke-Korinek M., Stanek G., Khanakah G., Kollaritsch H. (2007). Impact of a pertussis booster vaccination program in adolescents and adults on the epidemiology of pertussis in Austria. *The Pediatric Infectious Disease Journal*.

[9] Murphy T. V., Slade B. A., Broder K. R. (2008). Prevention of pertussis, tetanus, and diphtheria among pregnant and postpartum women and their infants recommendations of the advisory committee on immunization practices (ACIP). *MMWR. Recommendations and Reports: Morbidity and Mortality Weekly Report. Recommendations and Reports*.

[10] Gambhir M., Clark T. A., Cauchemez S., Tartof S. Y., Swerdlow D. L., Ferguson N. M. (2015). A change in vaccine efficacy and duration of protection explains recent rises in pertussis incidence in the United States. *PLoS Computer Biology*.

[11] Mansour-Ghanaei R., Moradi-Lakeh M., Shakerian S. (2016). Acellular pertussis vaccine efficacy: an updated systematic review and meta–analysis. *The Medical Journal of the Islamic Republic of Iran*.

[12] Centers for Disease Control and Prevention (2017). Prevention: Vaccine.

[13] van Boven M., Ruijs W. L. M., Wallinga J., O’Neill P. D., Hahné S. (2013). Estimation of vaccine efficacy and critical vaccination coverage in partially observed outbreaks. *PLoS Computational Biology*.

[14] Weinberg G. A., Szilagyi P. G. (2010). Vaccine epidemiology: efficacy, effectiveness, and the translational research roadmap. *The Journal of Infectious Diseases*.

[15] Central Statistical Agency (CSA) (2011). *Ethiopia Demographic and Health Survey from December 2010 to June 2011*.

[16] Amhara National Regional State Bureau of Health (2013). Health Research Thematic Areas of Amhara Regional Health Bureau: Amhara Regional Health Bureau Health Research and Technology Transfer Core Process in collaboration with Ethiopan Network for HIV/AIDS Treatment Care and Support (ENHAT-CS) Amhara Region Program.

[17] Roorda L., Buitenwerf J., Ossewaarde J. M., van der Zee A. (2011). A real-time PCR assay with improved specificity for detection and discrimination of all clinically relevant Bordetella species by the presence and distribution of three Insertion Sequence elements. *BMC Research Notes*.

[18] Murphy N. M., McLauchlin J., Ohai C., Grant K. A. (2007). Construction and evaluation of a microbiological positive process internal control for PCR-based examination of food samples for Listeria monocytogenes and salmonella enterica. *International Journal of Food Microbiology*.

[19] Juretzko P., von Kries R., Hermann M., Wirsing von König C. H., Weil J., Giani G. (2002). Effectiveness of acellular pertussis vaccine assessed by hospital‐based active surveillance in Germany. *Clinical Infectious Diseases*.

[20] Bentsi-Enchill A. D., Halperin S. A., Scott J., MacIsaac K., Duclos P. (1997). Estimates of the effectiveness of a whole-cell pertussis vaccine from an outbreak in an immunized population. *Vaccine*.

[21] Guiso N. (2009). Bordetella pertussisand pertussis vaccines. *Clinical Infectious Diseases*.

[22] Sheridan S. L., Frith K., Snelling T. L., Grimwood K., McIntyre P. B., Lambert S. B. (2014). Waning vaccine immunity in teenagers primed with whole cell and acellular pertussis vaccine: recent epidemiology. *Expert Review of Vaccines*.

[23] Simondon F., Preziosi M.-P., Yam A. (1997). A randomized double-blind trial comparing a two-component acellular to a whole-cell pertussis vaccine in Senegal. *Vaccine*.

[24] Baxter R., Bartlett J., Rowhani-Rahbar A., Fireman B., Klein N. P. (2013). Effectiveness of pertussis vaccines for adolescents and adults: case-control study. *BMJ*.

[25] Radke S., Petousis-Harris H., Watson D., Gentles D., Turner N. (2017). Age-specific effectiveness following each dose of acellular pertussis vaccine among infants and children in New Zealand. *Vaccine*.

[26] Nsubuga P., Eseko N., Tadesse W., Ndayimirije N., Stella C., McNabb S. (1998). Structure and performance of infectious disease surveillance and response, United Republic of Tanzania. *Bulletin of the World Health Organization*.

