# Erratum to “Assessment of Pertussis Vaccine Protective Effectiveness in Children in the Amhara Regional State, Ethiopia”

**DOI:** 10.1155/2020/8780407

**Published:** 2020-11-28

**Authors:** Solomon Taye, Belay Tessema, Baye Gelaw, Feleke Moges

**Affiliations:** ^1^Department of Medical Microbiology, School of Biomedical and Laboratory Sciences, College of Medicine and Health Sciences, University of Gondar, P.O. Box 196, Gondar, Amhara Regional State, Ethiopia; ^2^Department of Medical Laboratory Sciences, College of Medicine and Health Sciences, Wachemo University, P.O. Box 667, Hossana, South Nations Nationalities and Peoples Regional State, Ethiopia

In the article titled “Assessment of Pertussis Vaccine Protective Effectiveness in Children in the Amhara Regional State, Ethiopia” [1], author Solomon Taye was affiliated to “Department of Medical Laboratory Sciences, College of Medicine and Health Sciences, Wachemo University, P.O. Box 667, Hawassa, South Nations Nationalities and Peoples Regional State, Ethiopia,” which is incorrect.

The correct affiliation for this author is as follows:

^2^Department of Medical Laboratory Sciences, College of Medicine and Health Sciences, Wachemo University, P.O. Box 667, Hossana, South Nations Nationalities and Peoples Regional State, Ethiopia

The corrected list of affiliations is shown in the author information above.

This mistake occurred during the production of the article, and the publisher apologises for this error.
